# Predictive value of plasma galectin-3 levels in heart failure with reduced and preserved ejection fraction

**DOI:** 10.3109/07853890.2010.538080

**Published:** 2010-12-29

**Authors:** Rudolf A de Boer, Dirk J A Lok, Tiny Jaarsma, Peter van der Meer, Adriaan A Voors, Hans L Hillege, Dirk J van Veldhuisen

**Affiliations:** 1 Department of Cardiology, University Medical Centre Groningen, University of Groningen, PO Box 30 001, Hanzeplein 1, 9700 RB, Groningen, The Netherlands; 2 Department of Cardiology, Deventer Hospital, Deventer, The Netherlands

**Keywords:** Biomarkers, galectin-3, heart failure, prognosis, remodeling

## Abstract

**Aims.:**

Galectin-3 is an emerging biomarker which has been studied in relatively small heart failure (HF) cohorts with predominantly systolic HF. We studied the prognostic value of base-line galectin-3 in a large HF cohort, with preserved and reduced left ventricular ejection fraction (lvef), and compared this to other biomarkers.

**Methods.:**

We studied 592 HF patients who had been hospitalized for HF and were followed for 18 months. The primary end-point was a composite of all-cause mortality and HF hospitalization.

**Results.:**

A doubling of galectin-3 levels was associated with a hazard ratio (HR) of 1.97 (1.62–2.42) for the primary outcome (*P* < 0.001). After correction for age, gender, BNP, eGFR, and diabetes the HR was 1.38 (1.07–1.78; *P* = 0.015). Galectin-3 levels were correlated with higher il-6 and CRP levels (*P* < 0.002). Changes of galectin-3 levels after 6 months did not add prognostic information to the base-line value (*n* = 291); however, combining plasma galectin-3 and BNP levels increased prognostic value over either biomarker alone (RoC analysis, *P* < 0.05). The predictive value of galectin-3 was stronger in patients with preserved LVEF (*n* = 114) compared to patients with reduced LVEF (*P* < 0.001).

**Conclusions.:**

Galectin-3 is an independent marker for outcome in HF and appears to be particularly useful in HF patients with preserved LVEF.

## Introduction

Although progress has been made in early diagnosis and risk stratification of heart failure (HF), we are still short of tools to detect the disease early and to predict prognosis (1). Several biomarkers are used for diagnosis and prognosis of HF patients. Recently, galectin-3 has been proposed as a novel biomarker (2). Galectin-3 is secreted by activated macrophages and modulates several physiological and pathological processes (3) that contribute to HF, including inflammation and fibrosis. The up-regulation of myocardial galectin-3 has initially been demonstrated in a rat model of HF-prone hypertensive hearts (4). Subsequently, elevated levels of plasma galectin-3 in patients with acute (5) and chronic HF (6–8) were consistently associated with adverse outcome.

However, the studies published thus far only comprised several hundred patients. Clinical and biochemical correlates of galectin-3 are currently largely unknown. Furthermore, no data have been published on whether galectin-3 is a useful biomarker in patients with HF with preserved ejection fraction (HFPEF) in comparison with HF with reduced ejection fraction (HFREF). A recent analysis from the ProBNP investigation of dyspnea in the emergency department (PRIDE) (5) study suggested that galectin-3 particularly correlates with echocardiographic indices of *diastolic* function (9). Finally, the question as to whether serial measurements of galectin-3 have incremental value over base-line measurements alone has not been addressed.

**Key messages**

- Plasma galectin-3 is an independent predictor of outcome in large cohort of heart failure patients.
- The predictive power of plasma galectin-3 appears to be predominantly strong in heart failure patients with preserved left ventricular ejection fraction.
- Serial measurements of galectin-3 do not appear to add to the prognostic power of single measurements.

Therefore, we investigated the predictive value of galectin-3 in HF due to HFREF or HFPEF and compared this to an established biomarker, NT-pro-brain natriuretic peptide (NT-proBNP), and to several cytokines that have been associated with HF and that have been pathophysiologically related to galectin-3, including IL-6 and hsCRP. Finally, we evaluated if a follow-up measurement of galectin-3 taken after 6 months would further strengthen the value of galectin-3 in predicting outcome in HF.

## Methods

### Study design and outcome parameters

This is a prospectively designed substudy of the Coordinating study evaluating outcomes of Advising and Counseling in Heart failure (COACH) trial. The design and outcomes of the COACH trial (NCT98675639) have been published (10,11). Briefly, patients were included to participate in a prospective randomized disease management study. A total of 1,023 patients were included. Plasma for galectin-3 determination (and other biomarkers) was available from 592 patients during the index admission, and these are considered in the current subanalysis. Samples were collected just before discharge, when patients were stabilized after an acute HF admission. Mean follow-up was 18 months. Demographic and clinical data were collected during index admission from the medical charts. An additional plasma sample was taken after 6 months in 291 subjects during their visit to the outpatient department. To identify HFPEF, we chose a cut-off point of left ventricular ejection fraction (LVEF) > 40%, which is the same cut-off point as used in the CHARM trial (12).

for the current analysis, we used the primary outcome of COACH: time to first rehospitalization for heart failure or death. Hospitalization due to HF was defined as an unplanned overnight stay in the hospital due to worsening HF. Patients had to have typical symptoms and signs of HF, using standard criteria. All events were evaluated and adjudicated by an independent end-point committee. This study complies with the Declaration of Helsinki, local medical ethics committees approved the study, and all patients provided written informed consent.

### Measurement of galectin-3

The galectin-3 assay is an enzyme-linked immuno-sorbent assay (ELISA) developed by BG Medicine (BG Medicine, Inc., Waltham, USA). This assay quantitatively measures the concentration of human galectin-3 levels in EDTA plasma. This assay has high sensitivity (lower limit of detection 1.13 ng/ml) and exhibits no cross-reactivity with collagens or other members of the galectin family. Commonly used HF medication like angiotensin-converting enzyme (ACE) inhibitors, beta-blockers, spironolactone, furosemide, acetylsalicylic acid, warfarin, coumarines, and digoxin have no interference with the assay (13).

### Biochemical analysis of other of cytokines

Levels of cytokines (vascular endothelial growth factor (VEGF), interleukin-6 (IL-6), C-reactive protein (CRP), and transforming growth factor-β1 (TGF-β1)) in plasma samples were measured using Search-Light® Proteome Arrays (Aushon BioSystems, Billerica, MA, USA). The SearchLight Proteome Array is a quantitative multiplexed sandwich ELISA containing up to 12 different capture antibodies spotted on the bottom of a 96-well polystyrene microtiter plate. Each antibody captures a specific protein present in the standards and samples added to the plate. The bound proteins are detected with a biotinylated detection antibody, followed by the addition of streptavidin–horse–radish peroxidase (HRP), and lastly a chemiluminescent substrate. The luminescent signal produced from the HRP-catalyzed oxidation of the substrate was measured by imaging the plate using the SearchLight Imaging System which is a cooled charge-coupled device (CCD) camera. The data were analyzed using SearchLight Array Analyst software. The amount of luminescent signal produced is proportional to the amount of each protein present in the original standard or sample. Concentrations were extrapolated using a standard curve.

### Statistical analysis

We divided galectin-3 levels (ng/ml) in quartiles (1st quartile: 5.0–15.2; 2nd quartile: 15.2–20.0; 3rd quartile 20.0–25.9; 4th quartile 25.9–66.6). Base-line demographics are given in means ± standard deviation (SD) or as medians with interquartile ranges (IQR) when variables were non-normally distributed.

We conducted univariable analysis to evaluate the predictive value of galectin-3 (and other markers of prognosis) for the development of the primary outcome (composite of all-cause mortality and hospitalization due to HF). Then we conducted Cox regression analysis and stepwise included known predictors of prognosis to evaluate whether galectin-3 has independent prognostic value. If a factor modulated the predictive strength of galectin-3, we tested if interaction existed between the factors. We used receiver-operating characteristic (ROC) methodology to discriminate the predictive performance of different biomarker settings adjusted for age and sex. *P* values < 0.05 denote statistically significant differences.

## Results

### Study population

Base-line characteristics of the 592 patients in this subanalysis were comparable to those of the total COACH cohort (*n* = 1,023; data not shown). Mean age of the study population was 72 ± 12 years, and 65% were male patients. Half of the patients were in new york Heart Association (NYHA) class III and IV, the other half were in NYHA class II. LVEF was recorded mostly by echocardiography: mean LVEF was 0.33 ±0.15; 485 subjects had a LVEF ≤ 0.40, and 107 patients had a LVEF > 0.40. Mean estimated glomerular filtration rate (eGFR) was 55 mL/min/1.73 m^2^, median brain natriuretic peptide (BNP) value was 448 pg/mL, and patients were on standard medication for HF, including ACE inhibitors, beta-blockers, and diuretics.

### Base-line characteristics of patients stratified to galectin-3 levels (Table I)

Table I shows the base-line characteristics of patients according to quartiles of plasma galectin-3 levels. Patients with higher galectin-3 levels were older (*P* for trend < 0.001) and more often female (*P* = 0.023). Patients in NYHA class III and IV had higher galectin-3 levels (*P* < 0.001). BNP and NT-proBNP levels were also higher when galectin-3 levels were higher (*P* for trend 0.087 and < 0.001, respectively); however, absolute differences in BNP and NT-proBNP were rather small—patients in the lowest quartile of galectin-3 had a median BNP of 339 pg/mL and in the highest quartile 518 pg/mL. Patients with higher galectin-3 more often had diabetes and atrial fibrillation (*P* < 0.01). Treatment was comparable in all quartiles, except that patients in the highest quartile less often received ACE inhibitors (*P* = 0.002).

**Table I:** Base-line parameters according to the plasma galectin-3 levels.

	Quartiles of galectin-3 (ng/mL)				
		
Variables	Quartile 1 (5.0–15.2)	Quartile 2 (15.2–20.0)	Quartile 3 (20.0–25.9)	Quartile 4 (25.9–66.6)	*P*-value
*n*	148	148	148	148	
Age (years)	66 ± 11	70 ± 11	72 ± 11	76 ± 9	<0.001
Gender (% male)	69	64	62	52	0.023
NYHA (%, II / III / IV)	61/38/1	51/48/1	45/51/4	30/63/8	<0.001
BMI (kg/m^2^)	27 ± 5	28 ± 5	27 ± 5	28 ± 7	0.56
LVEF (%)	30 ± 13	34 ± 16	32 ± 15	34 ± 13	0.093
% patients LVEF ≥ 40%	18	28	23	26	0.26
eGFR (mL/min/1.73 m^2^)	67 ± 17	61 ± 18	50 ± 16	37 ± 15	<0.001
Hb (g/dL)	14.0 ± 1.9	13.3 ± 1.9	13.0 ± 2.0	12.5 ± 2.0	0.001
BNP (pg/mL) (median; IQR)	339 (173–780)	457 (190–781)	488 (244–1120)	518 (229–1240)	0.001
NT-proBNP (pg/mL) (median; IQR)	1767 (1048–3464)	2386 (1283–4666)	3051 (1480–6652)	4302 (1664–11640)	<0.001
Medical history (%)					
Hypertension	39	41	45	49	0.33
Myocardial infarction	38	39	42	43	0.74
Diabetes	19	29	37	35	0.004
Atrial fibrillation	35	41	49	57	0.001
COPD	22	28	26	36	0.048
CVA	7	12	9	13	0.35
Medication (%)					
ACE inhibitors	79	72	78	61	0.002
ARB	9	16	12	7	0.10
Beta-blocker	72	69	66	62	0.28
Diuretics	95	95	97	97	0.55

NYHA = New York Heart Association; Bmi = Body Mass Index; LVEF = Left Ventricular Ejection Fraction; eGFR = estimated glomerular filtration rate; Hb = hemoglobin; BNP = brain natriuretic peptide; NT-proBNP = NT-pro-brain natriuretic peptide; COPD = chronic obstructive pulmonary disease; CVA = cerebrovascular attack; ACE = angiotensin-converting enzyme; ARB = angiotensin receptor blockers.

### Galectin-3 and outcome

**Figure 1:**
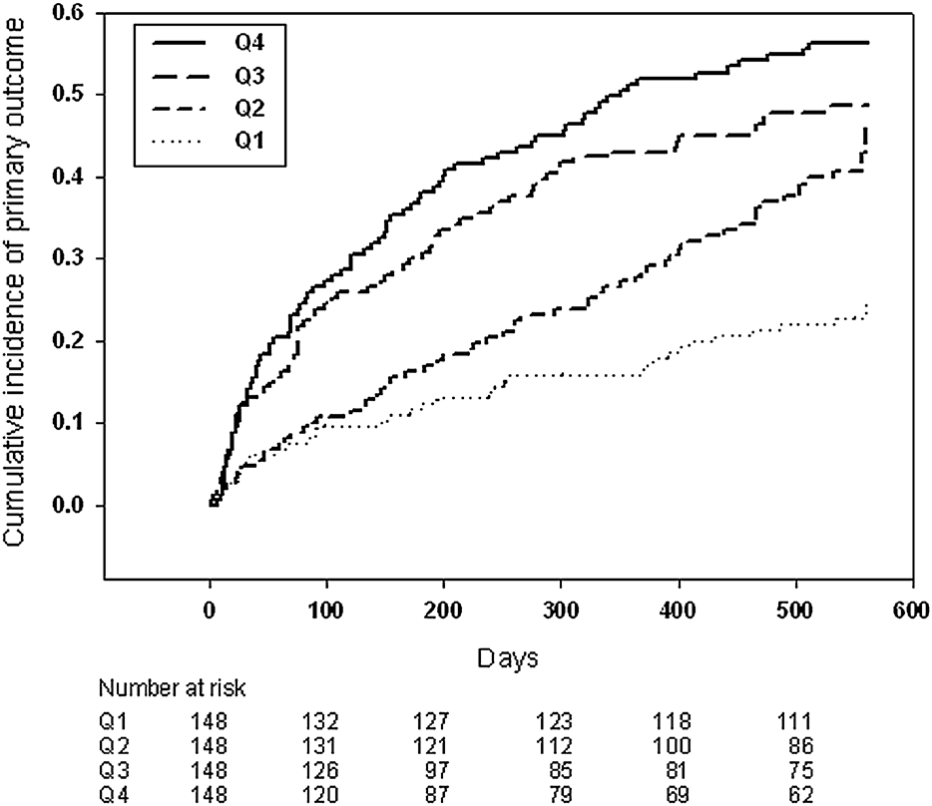
Adjusted Cox regression curves for quartiles of plasma galectin-3 showing the cumulative risk for the combined end-point all-cause mortality and hospitalization for HF.

*Primary outcome.* in a period of 18 months, 248 patients reached the primary outcome (164 deaths and 84 rehospitalizations due to worsening HF). Adjusted Cox regression curves for quartiles of galectin-3 are displayed in Figure 1. We conducted univariable analysis using patients in the lowest quartile of galectin-3 levels as a reference group (HR for the primary outcome set to 1.00). Compared to the reference group, patients in the second quartile had a hazard ratio (HR) of 1.98 (95% confidence intervals (CI) 1.29–3.02; *P* = 0.0016) for developing the primary outcome, patients in the third quartile a HR of 2.66 (95% CI 1.76–4.03; *P* < 0.001), and patients in the fourth quartile a HR of 3.34 (95% CI 2.23–5.01; *P* < 0.001). In Table II, it is shown that correction for age, gender, and BNP only marginally altered the predictive power of galectin-3. Correction for eGFR resulted in some loss, albeit very small, of predictive power of galectin-3, suggesting that some of the prognostic power of galectin-3 may be mediated via renal function. Correction for diabetes mellitus modestly mitigated the prognostic value of galectin-3. However, after correction for LVEF, plasma galectin-3 levels no longer statistically predicted the primary outcome. The interaction between LVEF and galectin-3 is described in the next paragraph.

**Table II:** Primary outcome: death or admission for heart failure: doubling of galectin-3.

Variable	Hazard ratio (95% CI)	*P* value
Galectin-3 (doubling)	1.97 (1.62–2.42)	<0.001
Adjusted for age (continuous), gender	1.90 (1.54–2.34)	<0.001
Adjusted for age (continuous), gender, BNP	1.77 (1.42–2.20)	<0.001
Adjusted for age (continuous), gender, BNP, eGFR	1.43 (1.11–1.85)	0.006
Adjusted for age (continuous), gender, BNP, eGFR, diabetes	1.38 (1.07–1.78)	0.015
Adjusted for age (continuous), gender, BNP, eGFR, diabetes, LVEF^a^	1.30 (0.97–1.74)	0.074
Interaction with LVEF^b^		0.047

a Continuous.

b Reduced LVEF (≤ 40%) versus preserved LVEF (> 40%).

Abbreviations as in Table I.

*Secondary outcomes: all-cause mortality and hospitalization due to HF.* We separately analyzed the individual elements of the primary end-point: 164 deaths (all-cause mortality) and 145 hospitalizations due to worsening HF (this number exceeds the number of hospitalizations (*n* = 84) of the primary outcome, as some patients were hospitalized several times). In the reference group (quartile 1), 34/148 patients experienced the primary outcome (23 deaths, 11 hospitalizations); in the 2nd quartile, 75/148 patients experienced the primary outcome (35 deaths, 40 hospitalizations); in the 3rd quartile, 94/148 patients experienced the primary outcome (42 deaths, 52 hospitalizations); and in the 4th quartile, 106/148 patients experienced the primary outcome (64 deaths, 42 hospitalizations). This subanalysis lacks power to provide significant differences in the secondary end-points after multiple correction (Supplementary Table I), although galectin-3 predicts mortality after correction for age, gender, BNP, and eGFR. Of note, the predictive value of galectin-3 was markedly less after correction for LVEF. The adjusted Cox regression curves for death and hospitalization due to HF, according to quartiles of galectin-3, are displayed in Supplementary Figures 1 and 2.

### Interaction between LVEF and prognostic value of galectin-3

**Figure 2:**
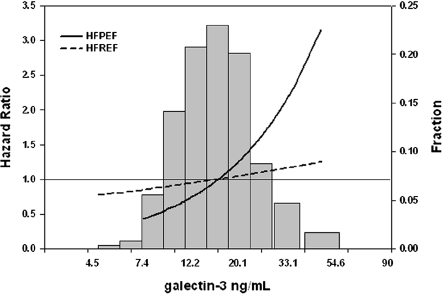
Graphical depiction of the risk estimates for experiencing the primary outcome in patients with HFPEF and HFREF with increasing levels of plasma galectin-3. The distribution of (log-transformed) galectin-3 is depicted in the background in brown bars. A similar increase in galectin-3 causes a much more pronounced increase in risk in patients with HFPEF compared to patients with HFREF.

We tested if LVEF has an interaction with the predictive value of plasma galectin-3. There was a statistically significant interaction between depressed LVEF (≤40%) and preserved LVEF (>40%) and the predictive value of plasma galectin-3 levels (*P* = 0.047). This interaction is graphically depicted in Figure 2. This figure shows that an identical increase in plasma galectin-3 levels represents a much stronger incremental risk for experiencing the primary outcome in patients with HFPEF in comparison with patients with HFREF (*P* < 0.001). However, *absolute* galectin-3 levels did not differ between patients with HFPEF and HFREF (Supplementary Table II). This table also shows the other characteristics of both groups: patients with HFPEF (*n* = 114) were older, were more often female, and had more often a history of hypertension, while plasma BNP was lower.

### Relation between galectin-3 and inflammatory cytokines (Table III and Table IV)

The cytokines VEGF, IL-6, and CRP had a significant positive correlation with plasma galectin-3 levels, although correlation coefficients were weak. To investigate if galectin-3 may operate in inflammatory response, we added the inflammatory cytokines to the multivariable model. None of the cytokines modulated the predictive power of galectin-3 on the primary outcome, although after correction of the full panel of cytokines the prognostic power of galectin-3 no longer reached statistical significance (*P* = 0.083) (Table IV).

**Table III:** Correlation and concentrations of four cytokines in relation to plasma galectin-3.

	Galectin-3 quartiles						
				
Analyte	Quartile 1 (5.0–15.2) (*n* = 148)	Quartile 2 (15.2–20.0) (*n* = 148)	Quartile 3 (20.0–25.9) (*n* = 148)	Quartile 4 (25.9–66.6) (*n* = 148)	*P* value^a^	Spearman's correlation coefficient	*P*-value Spearman
VEGF (pg/mL) (median; IQR)	57.1 (21.0–126.1)	57.3 (33.6–125.5)	61.2 (29.1–139.4)	76.1 (32.6–185.1)	0.043	0.1164 (*n* = 537)	0.0069
IL-6 (pg/mL) (median; IQR)	10.2 (4.7–20.0)	11.5 (7.6–19.4)	12.0 (6.6–26.5)	15.4 (8.8–30.3)	<0.001	0.1683 (*n* = 547)	0.001
CRP (mg/L) (median; IQR)	2.0 (0.5–3.9)	2.3 (1.0–4.1)	2.4 (0.9–6.5)	2.6 (1.3–6.1)	0.003	0.1354 (*n* = 547)	0.0018
TGF-β1 (ng/mL) (median; IQR)	46.6 (30.8–75.2)	50.7 (37.6–68.6)	51.4 (33.5–75.0)	51.0 (35.0–79.7)	0.172	0.0784 (*n* = 571)	0.0611

a kruskal–Wallis test for difference in medians across quartiles.

VEGF = vascular endothelial growth factor; IL-6 = interleukin-6; CRP = C-reactive protein;TGF-β1 = transforming growth factor-β1; IQR = interquartile range (25th percentile to 75th percentile).

**Table IV:** Primary outcome: death or admission for heart failure: doubling of galectin-3; influence of cytokines

Variable	Hazard ratio (95% CI)	*P* value
Galectin-3 (doubling), adjusted for age (continuous), gender, BNP, eGFR, diabetes (model 1)	1.38 (1.07–1.78)	0.015
Model 1 + CRP	1.36 (1.04–1.78)	0.026
Model 1 + IL-6	1.32 (1.01–1.71)	0.041
Model 1 + TGF-β1	1.35 (1.05–1.75)	0.021
Model 1 + VEGF	1.32 (1.02–1.72)	0.037
Model 1 + CRP, IL-6, TGF-β1, VEGF	1.29 (0.97–1.71)	0.083

Abbreviations as in Tables I and III.

### Comparison of galectin-3 with BNP as a biomarker in HF (Figure 3)

**Figure 3:**
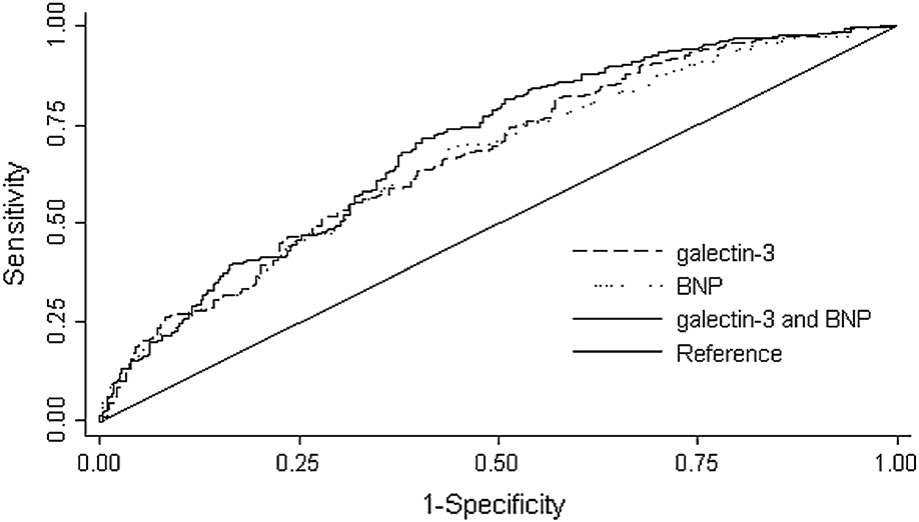
Combined receiver-operating characteristic (ROC) curves for brain natriuretic peptide (BNP) and galectin-3 for prediction of death or HF readmission in patients with HF after 18 months of follow-up. The ROC analysis for BNP showed an area under the curve (AUC) of 0.65 (*P* < 0.001); for galectin-3 the AUC is 0.67 (*P* = 0.004). The ROC analysis for the combination of BNP and galectin-3 shows an AUC of 0.69 (*P* < 0.05 versus BNP or galectin-3 alone).

We designed (age- and gender-adjusted) receiver-operating characteristic (ROC) curves to characterize further the value of galectin-3 in predicting the primary outcome. The ROC analysis of galectin-3 for the prediction of the primary outcome showed an area under the curve (AUC) of 0.67 (*P* = 0.004), while the AUC of BNP was 0.65 (*P* < 0.001). The combination of both galectin-3 and BNP was 0.69 (*P* < 0.05 versus BNP or galectin-3 alone) (Figure 3). Similar findings were observed with C-statistics using the bootstrap technique (data not shown).

### Value of repeated galectin-3 measurements (Figure 4)

Overall, plasma galectin-3 levels were very stable over a 6-month time period (data not shown). Differences over time in galectin-3 levels did not affect the power of base-line galectin-3 to predict outcome: after correcting the HR of ‘base-line galectin-3’ for the ‘6-month galectin-3’, base-line galectin-3 still powerfully predicted outcome (after correction for ‘6-month galectin-3 levels alone’: *P* < 0.001; or in combination with age, gender, BNP, and eGFR: *P* = 0.003). Furthermore, we designed combined ROC curves with both base-line galectin-3 and 6-month galectin-3, in order to assess if serial measurement of galectin-3 would yield superior predictive value compared to one single measurement at base-line. The ROC analysis for base-line galectin-3 for the prediction of the primary outcome showed an AUC of 0.67 (*P* = 0.004), while the AUC of 6-month galectin-3 was 0.66 (*P* = 0.04). The combination of both was 0.67 (*P* = NS versus base-line galectin-3 or 6-month galectin-3).

**Figure 4:**
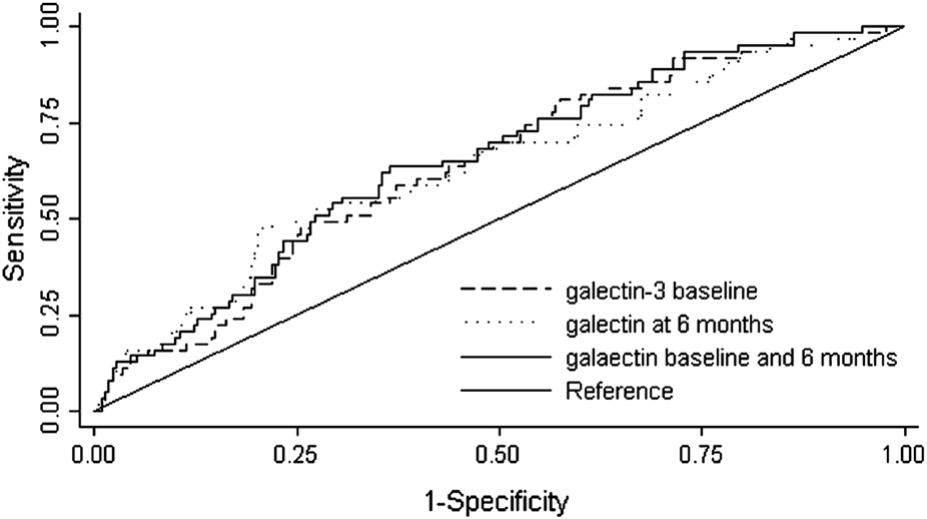
Combined receiver-operating characteristic (ROC) curves for the prediction of death or HF readmission in patients with HF after 18 months for galectin-3 levels at base-line and galectin-3 levels at 6-month follow-up. The ROC analysis for galectin-3 levels at base-line showed an area under the curve (AUC) of 0.67 (*P* for predicting the death or HF readmission: 0.004); the AUC for galectin-3 levels at 6 months is 0.66 (*P* = 0.04). The ROC analysis for a combination of galectin-3 levels at baseline and levels at 6 months follow-up showed an AUC of 0.67 (*P* = NS versus galectin-3 at base-line alone).

## Discussion

This is the largest study thus far in HF evaluating the predictive value of base-line plasma galectin-3 levels. We show that galectin-3 has independent prognostic value, even after correction for established risk factors for poor outcome in HF, including age, sex, BNP, renal function, and diabetes mellitus. In addition, we found an interaction with LVEF and plasma galectin-3 and report here that the prognostic importance of plasma galectin-3 levels appears to be much stronger in the subset of HF patients with preserved LVEF, in comparison to HF patients with reduced LVEF. Finally, base-line galectin-3 levels seem to suffice to predict outcome, as serial sampling did not increase the prognostic yield in our analysis.

Galectin-3 has recently been proposed as a useful biomarker involved in the pathophysiology of HF (2). Galectin-3 is widely distributed throughout the body, including expression in heart, brain, and vessels (3). Specifically, secretion of galectin-3 is associated with activation of fibroblasts and fibrosis (3). A rat model of HF revealed that galectin-3 is the strongest regulated gene when compensated left ventricular hypertrophy was compared with overt HF (4). The pathophysiological role for galectin-3 in development and progression of HF has been supported by several other experimental models of HF, like interferon-γ-induced murine chronic active myocarditis and cardiomyopathy (14), rat streptozotocin-induced diabetic cardiomyopathy (15), and angiotensin II-induced hypertension in rats (16).

Evidence from animal experiments has been supported by observations in humans. Galectin-3 was found to be significantly up-regulated in hypertrophied hearts of patients with aortic stenosis (4). Since galectin-3 levels can be reliably measured in plasma, several groups have explored the value of plasma galectin-3 as a biomarker in HF. However, until now only limited data are available in human HF. Our cohort is substantially larger than other published studies on galectin-3: van Kimmenade et al. (5) (acute HF), Lok et al. (6) (mild, chronic HF), Milting et al. (7) (end-stage HF), and Lin et al. (8) (chronic HF); and the COACH cohort was characterized by a very high event rate: in the current subset of 592 HF patients, 248 patients reached the primary end-point, and 164 died.

As previously observed (5,6), we found that the prognostic value of galectin-3 is independent from (NT-pro-) BNP levels. Natriuretic peptides are ‘loading markers’, which readily and strongly respond to ventricular stress, while galectin-3 levels is viewed as a marker of interstitial fibrosis, less responsive to (un-)loading. In support of this theory, Milting et al. (7) showed that unloading of poorly contractile hearts with assist devices causes a robust decrease in various neurohormones, including natriuretic peptides, but not galectin-3.

We describe for the first time that repeated measurements of galectin-3 levels (we measured at base-line and after 6 months, although in a small subset of patients, 291 out of 592) do not seem to add to the prognostic value. On an individual level, we observed that galectin-3 levels did not change substantially. This differs substantially from published observations with natriuretic peptides, which suggest that repeated measurement may increase diagnostic and prognostic yield and may be used to guide therapy (17,18). Arguably, galectin-3 activation and deposition in the matrix is an irreversible process and therefore less amenable to altered hemodynamics or other treatments (including pharmacological and nurse-lead intervention, like in the COACH trial). Possibly, specific anti-fibrotic treatment may negate the adverse effects of galectin-3 (16).

We furthermore evaluated if galectin-3 would have interaction with different pro-inflammatory cytokines, since it is well described that galectin-3 plays a central role in the inflammatory response, specifically in T cells (19–21).We correlated galectin-3 with an array of cytokines, which have been strongly linked to outcome in HF, like IL-6 and CRP (22,23). Overall, a significant trend was observed that with increasing galectin-3 levels pro-inflammatory cytokine levels also rise (Table III). When we correlated galectin-3 levels with the individual cytokines, however, correlation coefficients were weak. These data support the observation that galectin-3 may be involved in inflammation, also in HF. We hypothesize that given the modest correlation with cytokines, galectin-3 likely exerts its effects in HF predominantly via other pathways.

One of the most striking observations is that the predictive value of galectin-3 appeared to be stronger in patients with HF with preserved ejection fraction (HFPEF). Until now, all published data on the prognostic value of plasma galectin-3 levels were obtained in patients with HFREF (2,24). We defined HFPEF as LVEF > 40%, concurrent with the CHARM trial (11); however, other cut-off points have been proposed, like 45% (1) or 35% (25). Recently, a subanalysis from the PRIDE trial (5), involving 76 patients, showed that plasma galectin-3 strongly correlated with echocardiographic measurements of diastolic function (9). HFPEF is a very common entity with distinct features from HFREF (26,27). Few data are available on the use of biomarkers to diagnose HFPEF. Natriuretic peptides may be useful in this respect (28,29), but data are limited and by no means definite. Current guidelines (27) advocate the use of natriuretic peptides in the diagnostic work-up, but no other biomarkers are mentioned.

We observe an interesting interaction between LVEF and galectin-3. When we graphically explored the predictive value of galectin-3 in the patients with HFPEF versus HFREF (Figure 2) we found that an *identical* rise in galectin-3 levels is associated with a much stronger increase in the risk for reaching the primary end-point in patients with HFPEF (while average plasma galectin-3 levels did not differ substantially between patients with HFREF and HFPEF). In other words, in the current cohort, specifically in patients with HFPEF, increased galectin-3 levels are associated with worse prognosis. From the pathophysiology of HFPEF (26,27), which is characterized by hypertrophy, matrix apposition, and myocardial stiffening, it comes natural that a matrix and fibrosis marker like galectin-3 may be an important prognostic marker. Generally, HFPEF is more common in elderly, female patients and associated with more frequent co-morbidities such as hypertension and diabetes (26,27), which we confirm in our study (supplementary Table II). Previous observations (30) have indicated that other matrix proteins are also useful in this respect. Confirmation is needed in independent cohorts with HFPEF patients, but we postulate that galectin-3 might be a particularly useful biomarker in HFPEF. Of note, also in HFREF patients, increased levels of galectin-3 were associated with worse outcome in the COACH cohort, so that our current finding does not negate previous reports.

### Limitations

Plasma galectin-3 levels could only be measured in the subset of patients for whom base-line plasma levels were available, although the clinical characteristics of this subset did not differ from the entire COACH cohort. Sampling was at time of discharge so at variable time points, and at different levels of recompensation. Follow-up samples were available from a minority of the patients, which could have caused bias and decreases power with respect to the analysis of repeated galectin-3 sampling. We realize that the observation that galectin-3 may be particularly important in patients with HFPEF is limited by the small number of patients. The echocardiographic evaluation was not standardized to a protocol, and we have no other echo data than LVEF. Furthermore, new guidelines question the cut-off point of 40% to distinguish between HFPEF and HFREF. The findings should be regarded as exploratory and be confirmed in independent cohorts of patients with HF due to HFPEF. Finally, this analysis is underpowered to make decisive conclusions on the secondary end-points.

## Conclusions

In this to date largest HF cohort, we confirm that galectin-3 is a strong and independent prognostic factor. Inflammatory markers are positively correlated to galectin-3 levels. Repeated galectin-3 sampling has no incremental value over base-line sampling alone. Finally, galectin-3 might be a promising biomarker in patients with HFPEF which nowadays comprise about half of all HF patients.

## Figures and Tables

**Supplemental Table I:** Secondary outcomes: All cause mortality (death) and admission for heart failure: Doubling of Galectin 3

	Death (N = 164)		Hospitalizations due to Heart failure (N = 145)	
		
Variable	Hazard Ratio (95% CI)	*P*-value	Hazard Ratio (95% CI)	*P*-value
Galectin 3 (doubling)	2.15 (1.67 – 2.76)	<0.0001	1.76 (1.36 – 2.28)	<0.0001
Adjusted for age (continuous), gender	2.01 (1.54 – 2.61)	<0.0001	1.70 (1.30 – 2.22)	<0.0001
Adjusted for age (continuous), gender, BNP	1.74 (1.32 – 2.29)	<0.0001	1.65 (1.26 – 2.18)	<0.0001
Adjusted for age (continuous), gender, BNP, eGFR	1.42 (1.02 – 1.96)	0.036	1.26 (0.91 – 1.74)	0.162
Adjusted for age (continuous), gender, BNP, eGFR, diabetes	1.38 (1.00 – 1.91)	0.051	1.19 (0.86 – 1.65)	0.282
Adjusted for age (continuous), gender, BNP, eGFR, diabetes, LVEF*	1.21 (0.83 – 1.80)	0.320	1.00 (0.66 – 1.51)	0.991
Interaction with LVEF**		0.636		0.047

* continuous.

** reduced LVEFLVEF (≤40%) versus preserved LVEF (>40%).

**Supplemental Table II:** Patients characteristics from patients with HFREF and HFPEF.

Variables	HFREF LVEFLVEF ≤ 40%	HFPEF LVEFLVEF > 40%
N	368	114
Age (years)	69 ± 12	74 ± 10
Gender (% male)	66	50
NYHA (%, II / III / IV)	42/55/4	53/43/4
BMI (kg/m^2^)	26 ± 5	28 ± 6
LVEF (%)	26 ± 8	53 ± 8
eGFR (mL/min/1.73m^2^)	55 ± 20	53 ± 21
Hb (g/dL)	13.5 ± 1.9	12.6 ± 2.2
BNP (pg/mL) (median; IQR)	511 (232 – 1110)	310 (152 – 605)
NT-proBNP (pg/mL) (median; IQR)	3045 (1597 – 7278)	1898 (839 – 3827)
Galectin-3 (ng/mL) (median; IQR)	19.9 (14.8 – 25.7)	20.2 (16.0 – 26.0)
Medical history (%)		
Hypertension	40	51
Myocardial Infarction	44	30
Diabetes	28	29
Atrial Fibrillation	42	54
COPD	27	31
CVA	11	10
Medication (%)		
ACE inhibitors	77	58
ARB	10	16
Beta-blocker	72	60
Diuretics	96	95

## Supplemental material

*Supplementary material for* De Boer RA, Lok DJA, Jaarsma T, Van Der Meer P, Voors AA, Hillege HL, Van Veldhuisen DJ. Predictive value of plasma galectin-3 levels in heart failure with reduced and preserved ejection fraction. Ann Med. 2010;50:60–68.

Supplementary Figure 1 Adjusted cox regression curves for quartiles of plasma galectin 3 showing the cumulative risk for all cause mortality.

Supplementary Figure 2 Adjusted cox regression curves for quartiles of plasma galectin 3 showing the cumulative risk for rehospitalization due to worsening HF.

## Abbreviations

BNP: brain natriuretic peptide
COACH: Coordinating study evaluating outcomes of Advising and Counseling in Heart failure
eGFR: estimated glomerular filtration rate
HF: heart failure
HFPEF: heart failure with preserved ejection fraction
HFREF: heart failure with reduced ejection fraction
LVEF: left ventricular ejection fraction
NYHA: new york Heart Association
