# Supplementary figures and images for: Predictive value of plasma galectin-3 levels in heart failure with reduced and preserved ejection fraction
Source: Ann Med. 2010 Dec 29;43(1):60–8. doi: 10.3109/07853890.2010.538080 (PMC3028573; doi:10.3109/07853890.2010.538080)

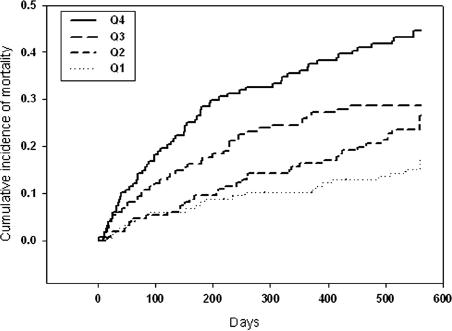

Supplement: Supplementary Figure 1 — Adjusted cox regression curves for quartiles of plasma galectin 3 showing the cumulative risk for all cause mortality. [file sann43-060-SD1.tif]

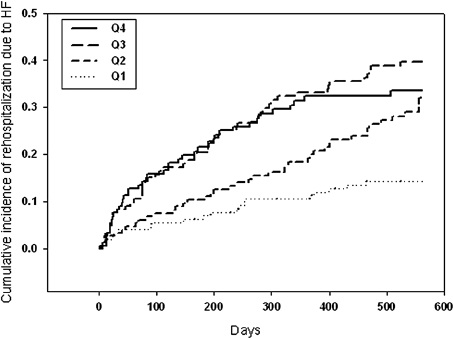

Supplement: Supplementary Figure 2 — Adjusted cox regression curves for quartiles of plasma galectin 3 showing the cumulative risk for rehospitalization due to worsening HF. [file sann43-060-SD2.tif]
